# Considerations in Using Artificial Intelligence to Simulate Orthodontic Treatment for Implant-Prosthetic Purposes

**DOI:** 10.3390/jcm15176813

**Published:** 2026-09-02

**Authors:** Amelia Smaranda Roșianu, Sanda Mihaela Popescu, Daniel Adrian Târtea, Stelian Mihai Sever Petrescu, Răzvan Mercuț, Marina Olimpia Amărăscu, Mihaela Ionescu, Ionuț Cristian Reşceanu, Alin Gabriel Ionescu

**Affiliations:** 1 Department of Oral Rehabilitation, University of Medicine and Pharmacy of Craiova, 200349 Craiova, Romania; amelia.popescu@umfcv.ro; 2 Department of Digital Dental Technology, University of Medicine and Pharmacy of Craiova, 200349 Craiova, Romania; adrian.tartea@umfcv.ro; 3 Department of Orthodontics, University of Medicine and Pharmacy of Craiova, 200349 Craiova, Romania; 4 Department of Plastic Surgery, University of Medicine and Pharmacy of Craiova, 200349 Craiova, Romania; razvan.mercut@umfcv.ro; 5 Department of Dental Technology, University of Medicine and Pharmacy of Craiova, 200349 Craiova, Romania; marina.amarascu@umfcv.ro; 6 Department of Medical Informatics and Biostatistics, University of Medicine and Pharmacy of Craiova, 200349 Craiova, Romania; mihaela.ionescu@umfcv.ro; 7 Department of Mechatronics and Robotics, Faculty de Automation, Computers, and Electronics, University of Craiova, 200440 Craiova, Romania; ionut@robotics.ucv.ro; 8 Department of History of Medicine, University of Medicine and Pharmacy of Craiova, 200349 Craiova, Romania; alin.ionescu@umfcv.ro

**Keywords:** preimplant orthodontic simulation, artificial intelligence, Medit Ortho Simulation, dentOne Autoalign

## Abstract

**Background/Objectives**: Orthodontic treatment planning software with artificial intelligence has been introduced into practice in recent years. They allow the simulation of the results of orthodontic treatment in several scenarios. The aim of the study was to compare the results of a simulated preimplantation orthodontic treatment plan in cases of oral rehabilitation between two software, with three different setups and their evaluation by an orthodontist. **Methods**: The in silico study included 10 cases of digital models obtained by intraoral scanning from patients with reduced lateral edentulousness who required implant-prosthetic treatment. The following software were used for the simulations: Medit Ortho Simulation v1.4.1 and Diorco dentOne v1.6.6.9 in two variants (Autoalign and a variant with fixation of the teeth bordering the edentulous breach). For comparison, the analysis of the Bolton report and of the movements in the three plans proposed by the software were used. **Results**: The results of the study showed that there were statistically significant differences between the simulations proposed by the two software tools. The evaluation made by the orthodontist showed that the Medit Ortho Simulation software was closer to the goal visualized by the orthodontist. **Conclusions**: The differences in movement between the simulations influenced the assessments of occlusions, tooth stability, and overall treatment planning that depend on the accuracy of vertical measurements.

## 1. Introduction

The development of digital dentistry has accelerated the use of intraoral scanners in clinical practice, in oral rehabilitation, in orthodontics, and in other dental domains. Digital impressions play a critical role in orthodontics and digital rehabilitation flows [1]. From digital models, the digitization of orthodontics has allowed the use of advanced planning tools. Current software platforms facilitate the simulation and three-dimensional visualization of treatment results, contributing to the realization and acceptance of the treatment plan [2,3]. These tools are based on digital models obtained by intraoral scanning. Compared to conventional impressions, intraoral scanning allows for faster data collection, increases patient comfort, and provides quick communication with the dental technician [2,4]. In orthodontics, the use of intraoral scanners supports the diagnosis and planning of orthodontic treatment through rapid data analysis, easy transfer of digital information, immediate access to patient records, and reduced need for physical storage [2,4]. As a consequence, the use of transparent aligners has become very common, as they are more compatible with digital flows compared to fixed orthodontic appliances [5]. The effectiveness of these therapies depends on rigorous digital analysis and planning, achieved through the integration of advanced model scanning and segmentation technologies [6,7].

The diagnosis and planning of orthodontic treatment as accurately as possible are both essential for the success of the final treatment. In this process, the analysis of study models plays an important role, including evaluations such as space analysis and Bolton analysis [8]. Space analysis is performed by comparing the sum of the mid-distal width of the teeth on an arch with the length of the arch, while Bolton analysis evaluates the ratio of sums of the mid-distal widths of the maxillary teeth compared to the mandibular teeth [9]. A Bolton discrepancy may occur when there are spaces, minor crowding, or edentulous spaces for an arch [10]. Identifying the excess or deficit of space with each arch allows for the orientation of the orthodontic corrections needed to achieve a functional occlusion [11]. Digital measurements of mesio-distal widths have comparable accuracy and repeatability to those obtained on conventional gypsum models [12,13]. Recent studies have indicated that systems powered by artificial intelligence (AI) may speed up data processing, improve decision accuracy, and support more effective therapeutic planning [5]. AI-based software platforms can create personalized treatment plans based on each patient’s specific anatomy and dental needs by evaluating large volumes of patient data and using machine learning algorithms to move teeth into the correct position [14,15]. AI systems are constantly learning and changing in response to real-world results, which helps them improve treatment plans and outcomes over time.

Digital dental models can be used to simulate an orthodontic treatment by separating individual teeth from dental models and aligning them in the desired position. The procedure known as “orthodontic teeth mounting” gives the orthodontist insights into the possibilities and limitations of the treatment, allowing the ultimate goal of the treatment to be visualized [2]. Orthodontic fitting of teeth can be useful in situations with intercalated edentulousness, as this requires decisions to be made in order to keep or to close the edentulous space. Digital setups based on intraoral impression data provide accurate information on changes in dental positions and arch shapes, produce 3D overlays for visualizing therapeutic changes, and facilitate the assessment of intra- and inter-arch relationships [16]. Multiple configuration variants can be generated and compared, supporting the choice of the final treatment plan. By segmenting and virtually repositioning the teeth in the desired position, digital models provide a configuration similar to the conventional one but made entirely in the virtual environment. After establishing the occlusal plane, each tooth can be repositioned by translation and rotation [17]. Numerous software programs are available for digital impression analysis and construction of these models, and their reliability has proven comparable to that of conventional models [3]. Auto-alignment software dentOne (Diorco Co., Yongin, Republic of Korea) automatically segments teeth and identifies the vestibular axis of the clinical crown corresponding to the most prominent portion of the central lobe on the vestibular surface of the incisors and premolars, namely the oral groove at the molar level [2]. After checking and adjusting this axis for each tooth, as well as defining the occlusal plane and the desired shape of the arch, the software automatically performs the dental alignment. Since the user must review the reference axes, establish the occlusal plane, and choose the shape of the arch before alignment, this type of program can be considered a semi-automatic configuration software [3]. Ortho Simulation (Medit, Seoul, Republic of Korea) is another example of automated configuration software. Similar to other software applications, it is integrated into the Medit intraoral scanning software, and exclusively supports scans obtained with Medit intraoral scanners. After importing the data, the clinician defines the upper and lower dental midlines, and the software automatically identifies the teeth and generates the digital setup [18].

As AI technologies advance, digital setup programs are increasingly automating previously manually performed steps, including dental segmentation and alignment [19]. The evaluation of the quality of models generated by fully automated configuration was done by comparing the accuracy and efficiency of automated software with the manual digital configuration models made by the clinician [3]. Although a recently published analysis highlighted the rapid development of artificial intelligence in orthodontics, its limitations persist despite the increase in the number of studies and recent technological advances [20].

To date, the performance of automatic configuration software has not been sufficiently evaluated. AI-assisted treatment planning, the assessment of maxillary and mandibular dental arch shapes, and the assessment of the space required to manage reduced intercalated edentations and dental crowding reflect insufficiently investigated areas that require studies with innovative methodologies [6,21,22].

The aim of this study was to compare the proposed setups for orthodontic treatment in patients with intercalated edentulousness, as well as the results of Bolton analysis, between two types of AI-based software. The null hypothesis was that the two software programs would perform the same type of therapeutic setup. The alternative hypothesis was that AI-based orthodontic simulation tools would show statistically significant differences between therapeutic setups.

## 2. Materials and Methods

This in silico study was performed on intraoral scans obtained from a group of 10 patients aged 23–30 years with reduced lateral edentulousness, which were from the archive of intraoral scans of the Oral Rehabilitation Discipline of UMF Craiova. The digital models were obtained through intraoral scans acquired with the Medit I700 scanner (Medit, Seoul, Republic of Korea) made for implant-prosthetic treatment planning. The files scanned in .obj format were then used for orthodontic analysis and orthodontic planning in two commercial software: Medit Ortho Simulation v1.4.1 (Medit, Seoul, Republic of Korea) and dentOne software v1.6.6.9 (DIORCO Co., Ltd., Yongin, Republic of Korea).

Thus, three simulations were obtained in each case: a fully automatic simulation performed by Medit Ortho Simulation, with an implant proposal instead of edentulousness; a simulation performed by dentOne Autoalign; and a simulation performed by dentOne, with the option to maintain the same positions of adjacent teeth in the edentulous space, as this software does not have the ability to take into an account an implant instead of an edentulous space.

### 2.1. Performing the Simulation of the Orthodontic Treatment Plan Before Implant-Prosthetic Rehabilitation with Medit Ortho Simulation

The simulation of creating the implant space within the orthodontic treatment project, carried out with the Medit Ortho Simulation application from the Medit Link platform v3.4.7, was performed using the information extracted from the .stl files obtained after intraoral scanning with the Medit I700 scanner (Medit, Seoul, Republic of Korea). Subsequently, it was processed using the Medit Scan for Clinics application v1.13.x (the software program for acquiring and processing scanned images).

Medit Ortho Simulation’s AutoAlign feature is an AI-driven orthodontic alignment system that processes intraoral scans through a structured sequence of digital operations —noise reduction, gingival edge detection, and individual tooth segmentation—before analyzing the geometric features of dental arches, including the occlusal plane, midline, curvature, and bilateral symmetry, to establish a mathematical frame of reference for the optimal positions of teeth. In this computational environment, the algorithm repositions each tooth to reduce rotation, crowding, and spacing while maintaining realistic interproximal contacts, thereby preventing volumetric collisions by relying on a collision-conscious optimization routine that preserves anatomical plausibility. When the insertion of an implant into an edentulous space is anticipated, Ortho Simulation introduces a generic cylindrical substitute that functions exclusively as a spatial constraint, ensuring that the AutoAlign process does not collapse or distort the restorative space. Although this temporary element is not anatomically calibrated for any implant system, it effectively stabilizes the mid-distal site boundaries during alignment. As the system harmonizes the upper arch with the lower arch through occlusal contact stabilization and inter-arch geometric balancing, the end result is a symmetrical and diagnostically coherent digital arrangement with no collisions that supports orthodontic visualization, aligner simulation, restorative planning, and interdisciplinary communication, all while retaining the biomechanical design of orthodontic treatment or specific surgical planning of the implant.

**Figure 1:**
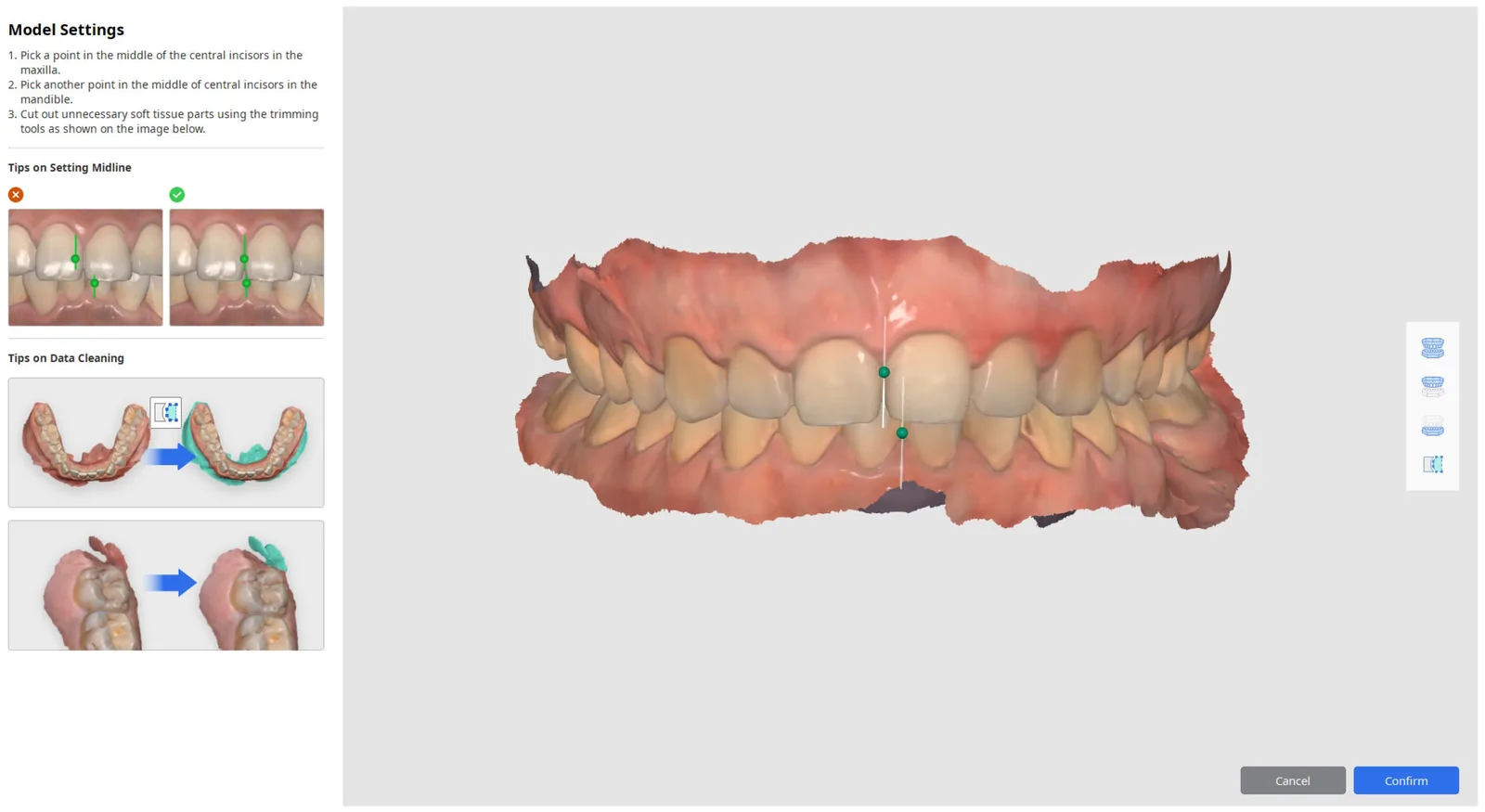
Model alignment in Medit Ortho Simulation app.

**Figure 2:**
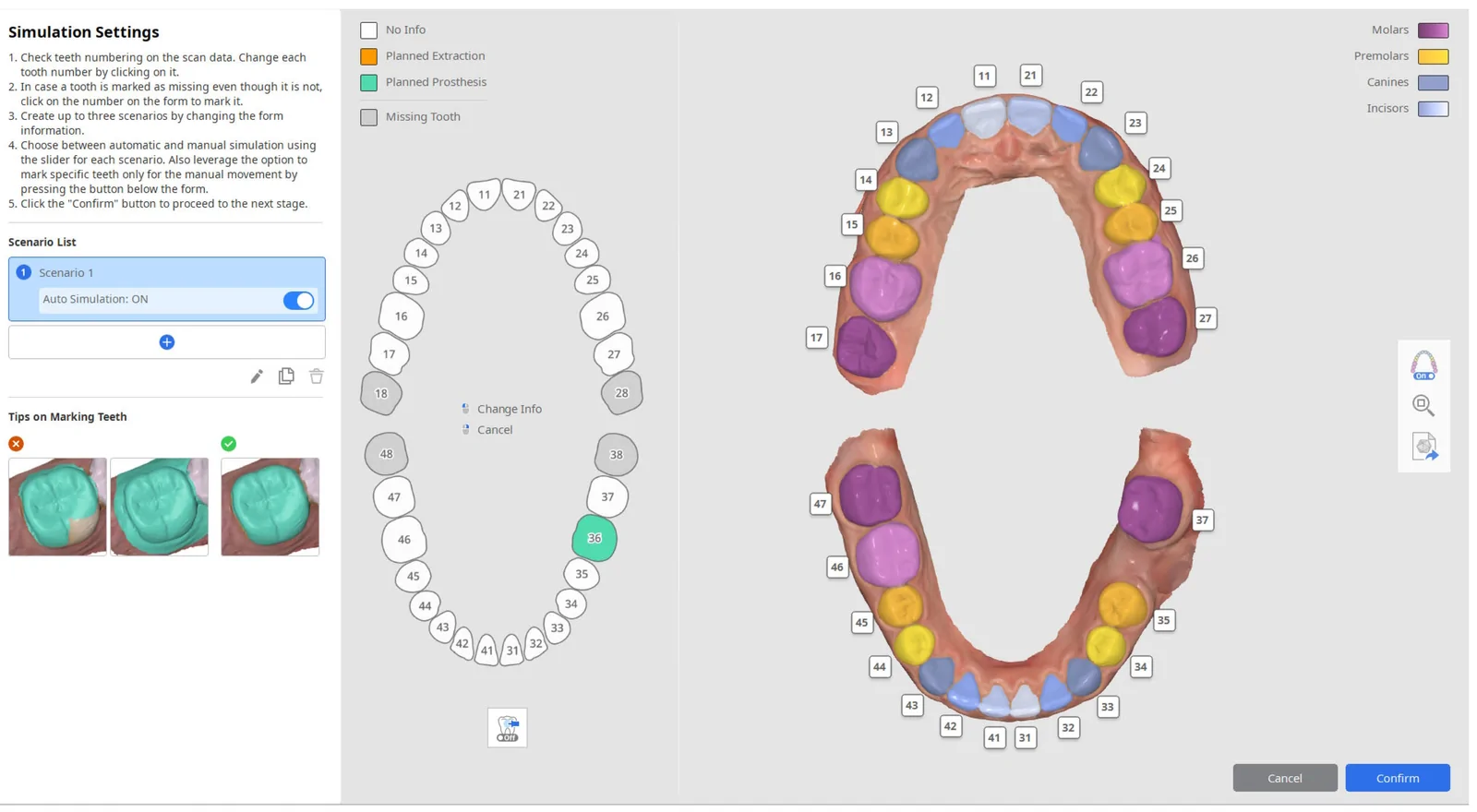
Model segmentation in the Medit Ortho Simulation app.

**Figure 3:**
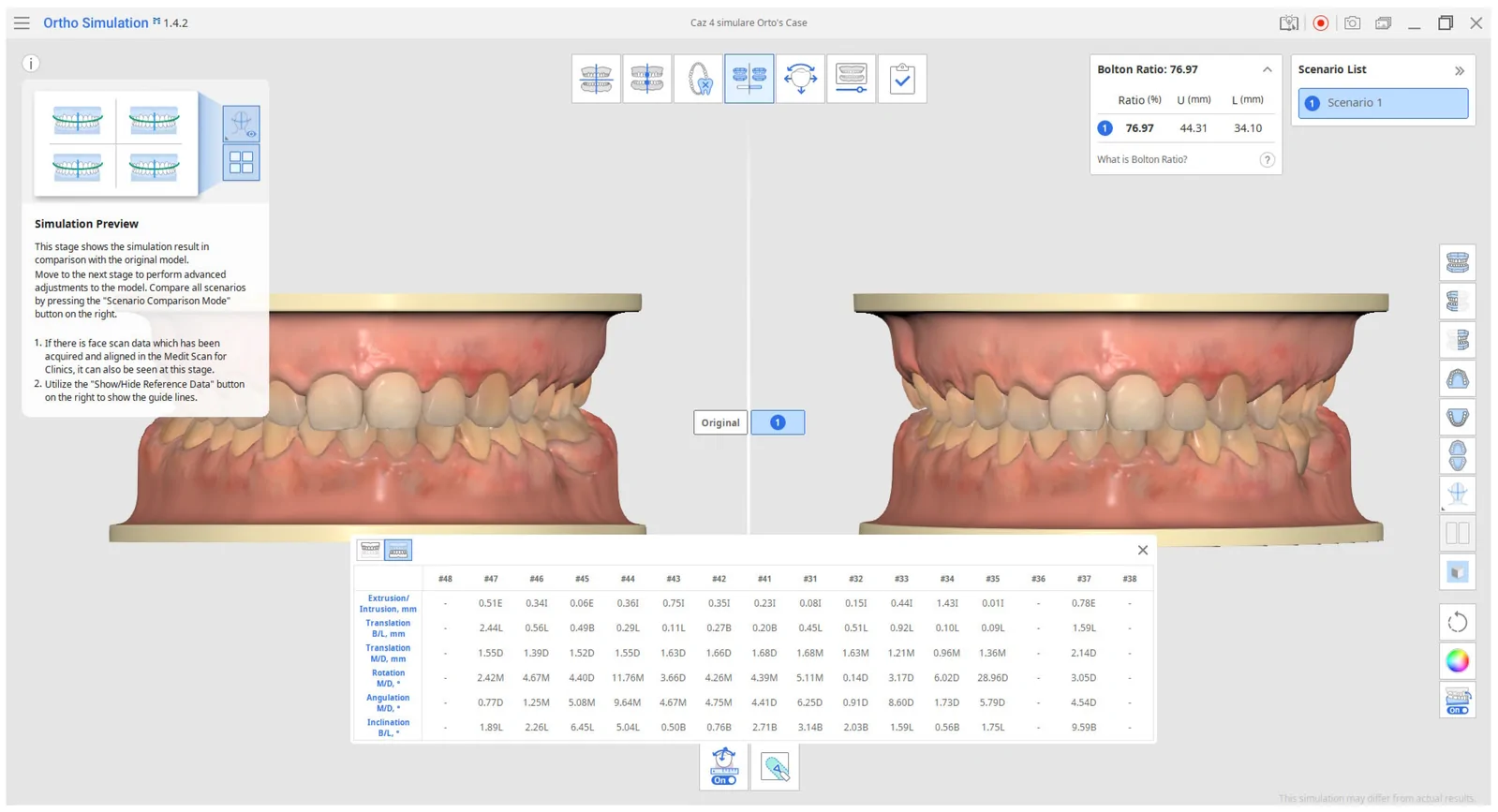
Orthodontic project simulation in the Medit Ortho Simulation app.

For each case, images of the scanned maxillary and mandibular arches and of the two arches in occlusion were used. The necessary steps for the orthodontic project were: opening of the application and loading the data of the two arches and model settings (removal of imperfections, orientation of the maxillary and mandibular arches, definition of the midline and the occlusion plane) (Figure 1), definition of the treatment scenario in which the placement of the crown on the implant was indicated, segmentation and teeth numbering performed by the software application (Figure 2), their verification and confirmation by the operator, followed by the completion of the final orthodontic project (Figure 3).

Subsequently, numerical data were extracted regarding the displacement of teeth in three planes (sagittal, transverse, vertical) in the form of vestibulo-oral translation, mesio-distal translation, and vertical movement of intrusion–extrusion type, as well as the displacement in another three directions—rotation, inclination, and angulation—by the inclination relative to the reference planes (vertical, sagittal, transversal).

### 2.2. Performing the Simulation of the Orthodontic Treatment Plan Before Implant-Prosthetic Rehabilitation with dentOne Autoalign

The simulation of the orthodontic treatment project carried out with the dentOne Autoalign application (DIORCO or “Digital Orthodontic Company”, 184 Jungbu-daero, Giheung-gu, Yongin-si, Gyeonggi-do, Republic of Korea) was performed based on the data received from the .obj files obtained after intraoral scans with the Medit I700 scanner (Medit, Seoul, Republic of Korea) and processed with the Medit Scan for Clinics application.

The dentOne AutoAlign feature is an algorithmic alignment system that repositions teeth into an ideal arch configuration by integrating geometric analysis, dental repositioning, and orthodontic rules. After preparing the 3D models, the software segments the teeth and turns each crown into an independently mobile digital object. Subsequently, the shape of the arch, midline, occlusal plane, and bilateral symmetry are analyzed in order to establish a mathematical reference curve that guides alignment. Based on this, AutoAlign calculates the optimized positions for each tooth by reducing rotations, leveling the occlusal plane, normalizing spacing, and distributing crowding along the arch while avoiding volumetric intersections between teeth. The result is a harmonized, collision-free, and geometrically coherent dental arrangement that is useful for diagnostic visualization, digital setups, and workflows with transparent aligners, though this does not represent a complete orthodontic biomechanical planning.

**Figure 4:**
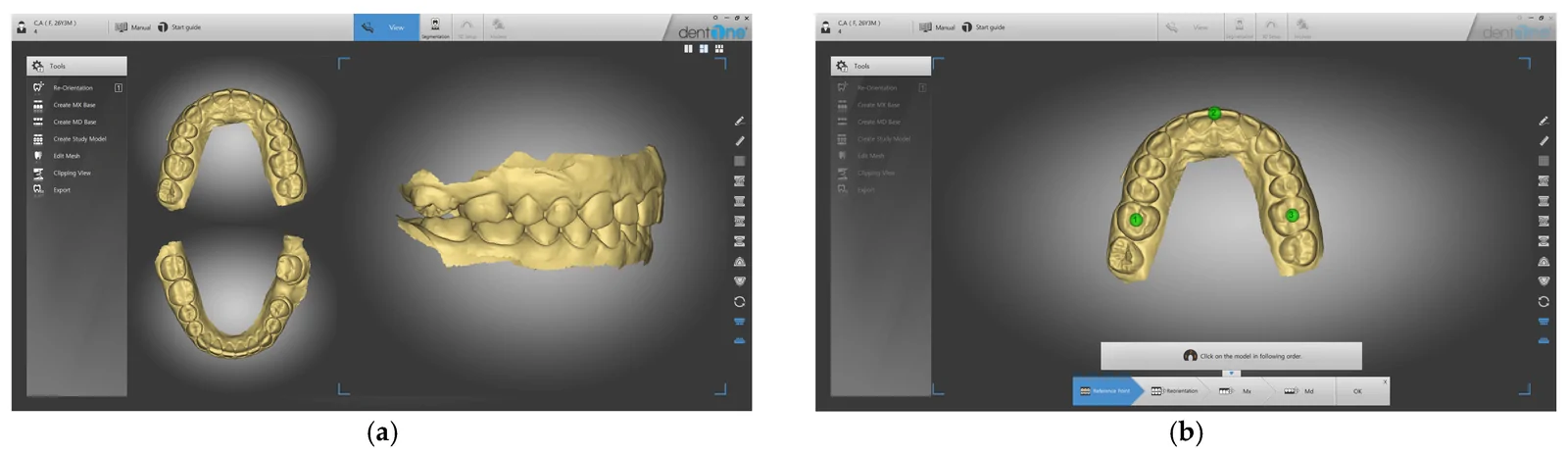
Pre-established steps for using the dentOne design software: (**a**) uploading models to the dentOne app; (**b**) setting model benchmarks.

**Figure 5:**
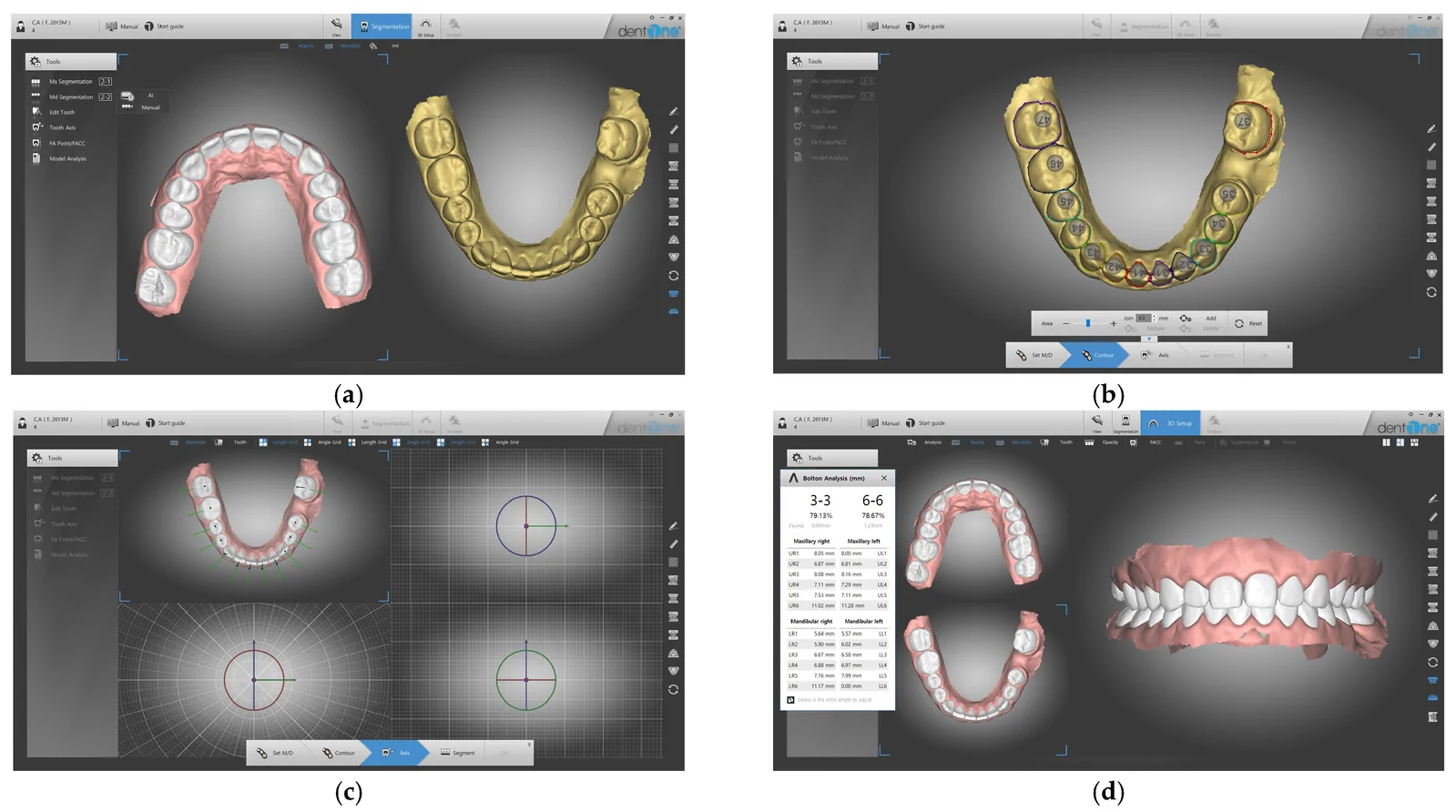
Steps when using dentOne app for case evaluation: (**a**,**b**) model segmentation in the dentOne app; (**c**) identification of mandibular tooth axes with AI; (**d**) initial Bolton report in the dentOne app.

For each case, images of the scanned maxillary and mandibular arches and of the two arches in occlusion were used. The necessary steps for the realization of the orthodontic project were: opening the online platform and uploading the data of the two arches and all the data related to each case. From the online platform, the dentOne design software is accessed by following pre-established steps, starting with the model settings (orientation of the maxillary and mandibular arches, definition of the midline and the occlusion plane) (Figure 4a,b), followed by the segmentation and automatic teeth numbering, identification of the axes of the mandibular teeth with AI (Figure 5a–c), resulting in an initial Bolton ratio (Figure 5d).

**Figure 6:**
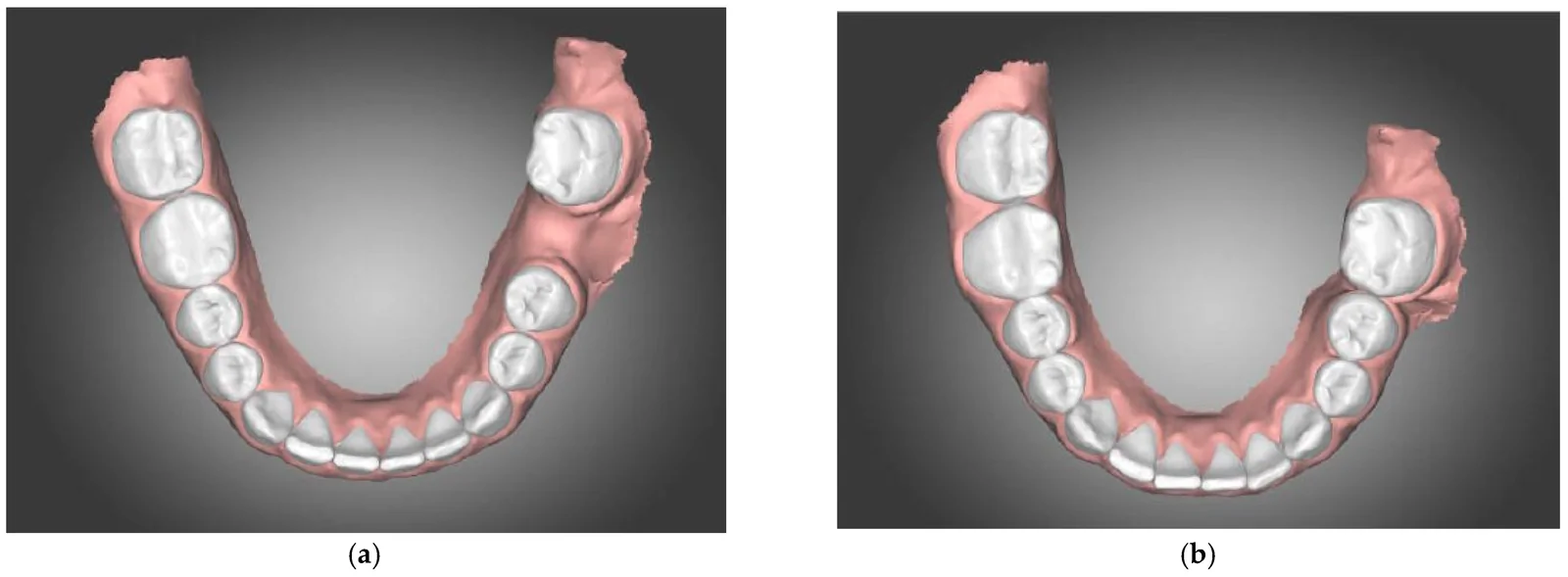
DentOne software used to simulate the management of tooth loss by closing the space: (**a**) edentulous space in 36; (**b**) closing edentulousness 36 with the Autoalign function of dentOne software.

The dentOne Autoalign function in the 3D Setup stage closes all detected edentulousness so that the edentulous 36 disappears with a distal tilt of 35 and a mesial tilt of 37 (Figure 6a,b).

Subsequently, numerical data were extracted regarding the displacement of teeth in three planes (sagittal, transverse, vertical) in the form of vestibulo-oral translation, mesio-distal translation, and vertical movement of intrusion–extrusion type, as well as inclination in three directions (rotation, inclination, angulation) relative to the reference planes (vertical, sagittal, transversal).

### 2.3. Realization of the Simulation of the Orthodontic Treatment Plan Before Implant-Prosthetic Rehabilitation with dentOne with Fixed Adjacent Teeth

To maintain space for a future implant, the dentOne app preserves an edentulous space for a future implant through a constraint-based alignment mechanism, in which the clinician manually sets the desired meso-distal boundaries of the implant site and then fixes the adjacent teeth so that AutoAlign treats their positions as immobile reference anchors. The algorithm uses constraints that respect the fixed teeth, including teeth adjacent to a future implant space, so that the protected areas remain stable during alignment. When the clinician manually defines a target space—for example, for implant planning or to provide a specific restorative space—dentOne preserves this configuration by locking the relevant teeth before applying the AutoAlign function. After the initial scan is segmented and each tooth is transformed into an independently manipulable 3D object, the clinician adjusts the mesial–distal translation of the teeth adjacent to the edentulous area until the target implant space is reached, measured, and verified using dentOne’s built-in distance tools. Once these edge teeth are positioned correctly, dentOne’s Fix Tooth feature is applied, turning them into rigid constraints within the alignment algorithm. During the AutoAlign process, the software analyzes the shape of the dental arch, occlusal plane, midline, and symmetry to calculate optimized positions for all non-set teeth, but it explicitly excludes fixed teeth from translational or rotational adjustments. As a result, the algorithm preserves the exact width and location of the implant site while performing global alignment, leveling, and spacing corrections on the remaining dentition. This approach ensures that the final digital configuration maintains an adequate clinical restorative space, prevents collapse of the edentulous region, and produces a coherent geometric arch shape suitable for implant planning, restorative design, and orthodontic simulation, all while remaining distinct from the complete planning of biomechanical orthodontic treatment.

The simulation of creating the implant space within the orthodontic treatment project, carried out with the dentOne application (in the 3D Setup stage with the blocking of adjacent teeth), was performed based on the information received from the obj. files obtained after intraoral scans with the Medit I600 scanner (Medit, Seoul, Republic of Korea) and processed using the Medit Scan for Clinics application.

**Figure 7:**
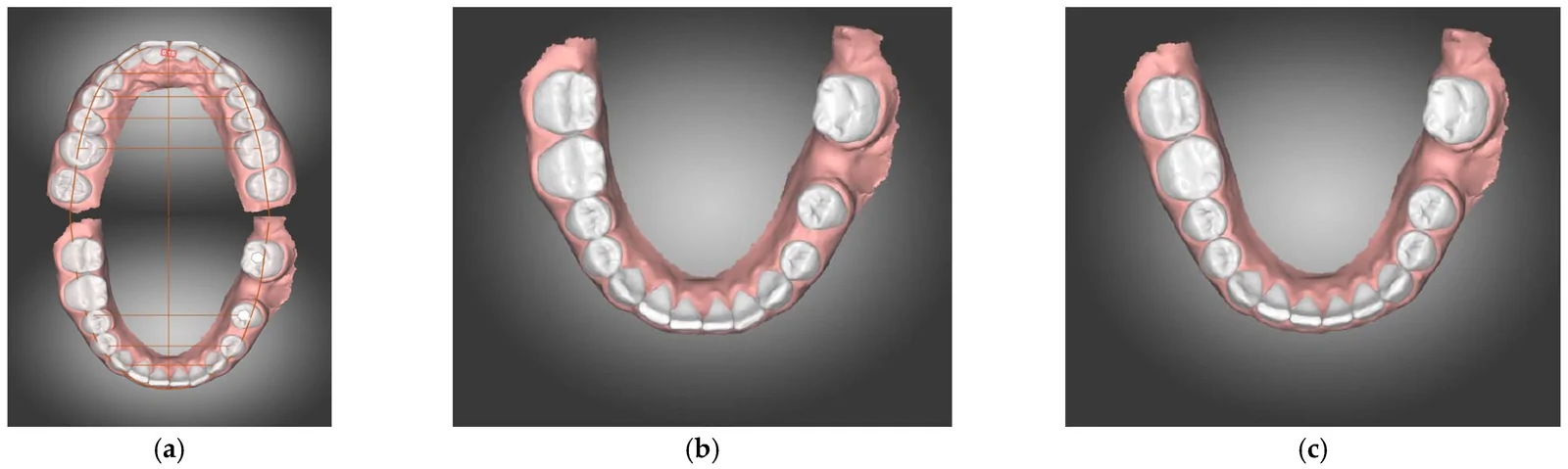
DentOne app prepared for simulation by locking the positions of neighboring teeth: (**a**) locking teeth 35 and 37 with Tooth Setup function; (**b**) initial model; (**c**) final model.

The first necessary steps for the orthodontic project were the same as those presented in the case of dentOne Autoalign. The differences emphasize the special 3D Setup function of the application, with the possibility to block teeth bordering the edentulous space, as the software does not have a specific function related to the planning of a dental implant. The Tooth Setup function still allows the blocking of bordering teeth, so that the edentulousness 36 remains in the real dimensions from the intraoral scan, but blocks any change in the positions of teeth 35 and 37. By using this function to maintain the positions of adjacent teeth, the software achieves an automatic alignment in compliance with these restrictions, resulting in a mandibular model aligned with an edentulous space 36 that can be prosthetized by inserting a dental implant (Figure 7a–c).

Subsequently, numerical data were extracted regarding the displacement of teeth in three planes (sagittal, transversal, vertical) in the form of buccal–oral translation, mesio-distal translation, and vertical movement of intrusion–extrusion type, as well as inclination in three directions (rotation, inclination, angulation) relative to the reference planes (vertical, sagittal, transversal).

### 2.4. Evaluation Performed by an Orthodontist of the Simulations of Orthodontic Treatment Plans in the Three Setups

Following the completion of the three simulations for the treatment plans, an orthodontist from the team evaluated the correctness of the treatment plans. The orthodontist rated the software planning performances with grades between 1 (the lowest) and 10 (the highest), providing arguments for each decision.

### 2.5. Analysis of the Bolton Ratio

The Bolton value reflects the ratio between the sum of the widths of the mandibular teeth and the sum of the widths of the maxillary teeth, measured mesial–distal, representing an indicator that aims to assess the dimensional balance between the teeth on both arches. Its value indicates the presence of an excess of teeth size in one of the arches compared to the other, which directly influences the occlusion, spacing, and alignment of the teeth.

The Bolton ratio expresses two calculation variants for two different sets of segments, indicating the degree of fit of the two arcades.

- Anterior Bolton ratio (3–3)—Compares the 6 front teeth (incisors and canines); ideal value: 77.2% (±1.65%).
- Total Bolton ratio (6–6)—Compares 12 teeth (from the right first molar to the left first molar); ideal value: 91.3% (±1.91%).

If the computed value of this ratio is higher than the ideal value, it reflects a mandibular excess (the lower teeth are large). If the ratio value is lower than the ideal value, it reflects a maxillary excess (the upper teeth are large). The difference is expressed in mm of dental substance that must be adjusted by various dental procedures (IPR, build-ups, restorations).

The calculation of the Bolton ratio involves the presence of all maxillary and mandibular teeth. If one or more teeth are missing from the arches, the calculation of the Bolton ratio involves the use of substitute values for each missing tooth. The most commonly used method substitutes the width of the missing tooth with the value of the width of its contralateral. If the lack of teeth is bilateral, the mean anatomical values are used instead. In cases where the treatment plan includes the intention to insert an implant or a bridge, the planned restoration size shall be used.

For the manual calculation of the Bolton ratio for each clinical case in the study group, the substitution method was used with the value of the contralateral width of all missing teeth.

### 2.6. Statistical Analysis

The data from Medit Ortho Simulation and dentOne application were initially processed using the Microsoft Excel 365 software application (San Francisco, CA, USA) to centralize the summary tables and graphs. Subsequently, the measurements were analyzed using the Statistical Package for Social Sciences (SPSS), version 26 (IBM Corp., Armonk, NY, USA). Continuous variables were reported as percentages (for the Bolton ratio), or as a pair of “mean ± standard deviation (SD)” and median values. The normality of the continuous data series was analyzed using the Shapiro–Wilk test, and the homogeneity of the variations was analyzed with the Levene test. Based on these results, group comparisons for all continuous variables were performed using the Mann–Whitney U or Kruskal–Wallis H tests (followed by Dunn’s procedure for post hoc analysis, if necessary). Variations in measurements were analyzed using the Wilcoxon signed-rank test (when the differences between measurements made before and after simulating the orthodontic treatment plan were not normally distributed). A 5% threshold was considered to be statistically significant, so the *p*-value < 0.05 was defined as the threshold for interpreting the results of the statistical tests.

## 3. Results

The analyzed cases presented one or more maxillary and/or mandibular lateral edentulations, with the inclination of teeth adjacent to the spaces (Table 1).

**Figure 8:**
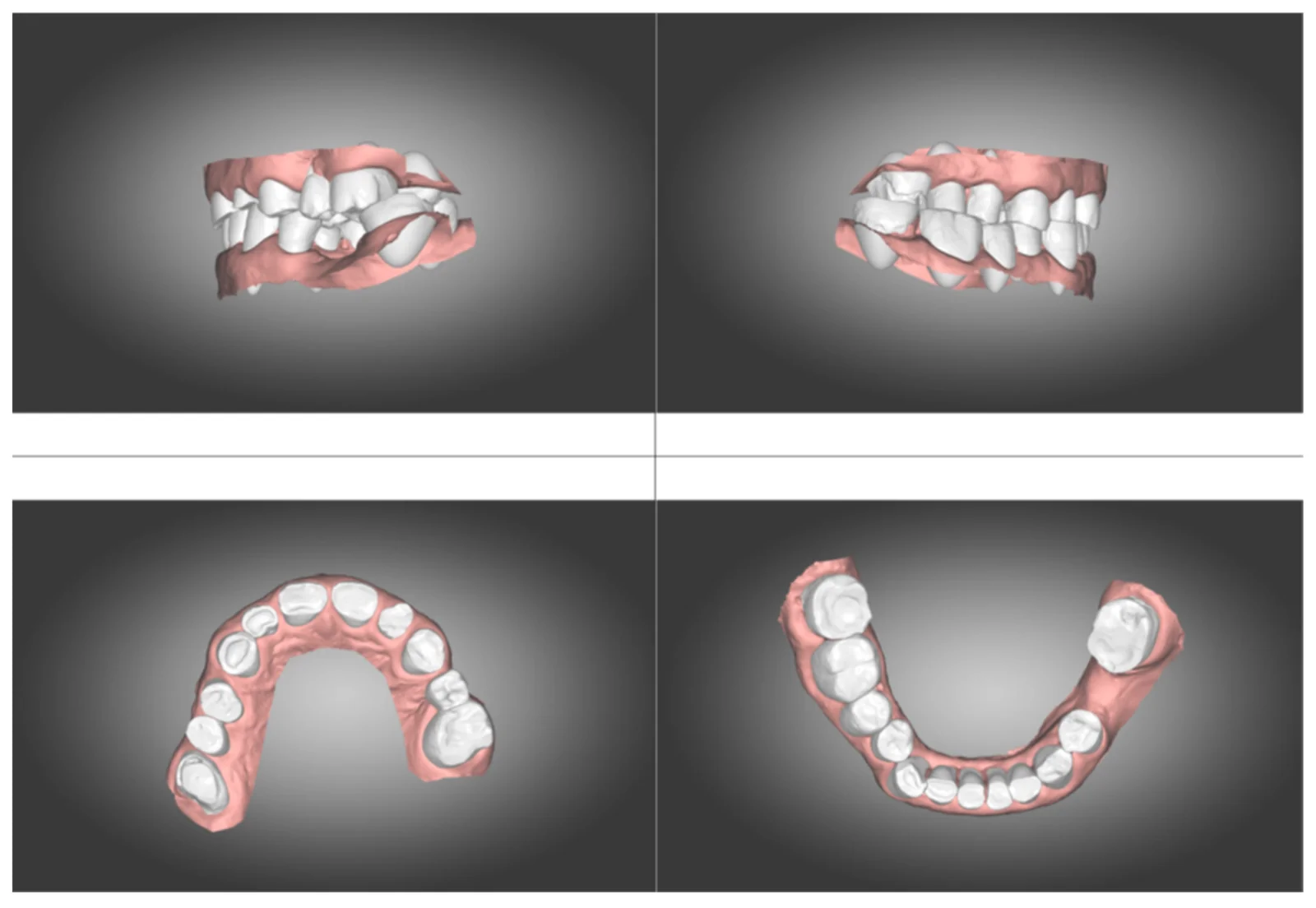
An example of a case with a lower-jaw single edentulism that was used when testing the simulations from the two apps.

Case example: Case 1—Tooth loss of 36 with the distal inclination of 35 and the mesial inclination of 37; absence of 27 (Figure 8).

### 3.1. Bolton Ratio Analysis

**Figure 9:**
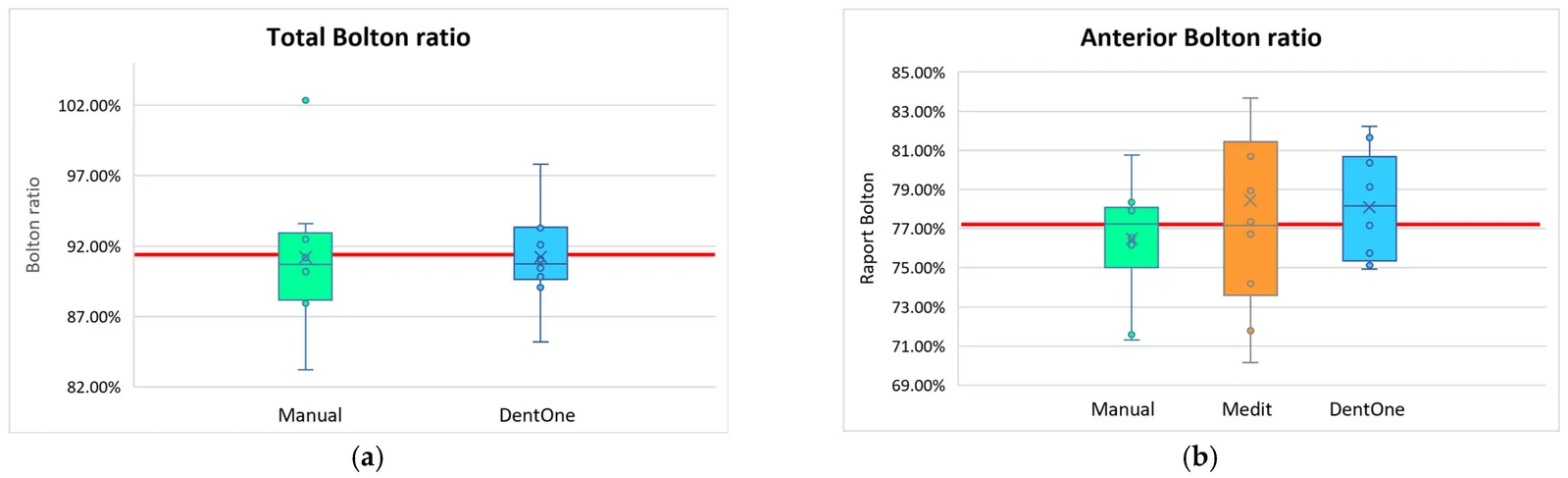
Bolton ratio value ranges for patients included in the study group: (**a**) total Bolton ratio; (**b**) anterior Bolton ratio. For each chart, the red line represents the values considered optimal for the Bolton ratio (total and anterior).

The total Bolton ratio values, measured manually, ranged from a minimum of 83.20% to a maximum of 102.35%, with an average of 91.21% ± 4.94% and a median value of 90.71% slightly below the optimal value, suggesting a balanced overall distribution. The same data provided by dentOne had a median value of 90.75%, with a lower dispersion and a compact distribution, indicating a relatively good compatibility between the dental dimensions of the two arches but also a tendency to get closer to the theoretical ideal threshold (Figure 9a).

The anterior Bolton ratio values, measured manually, ranged from a minimum of 76.16% up to the highest value of 80.76%, with an average of 76.48 ± 2.97% and a median value of 77.23%, very close to the ideal value of 77.20%. The anterior Bolton ratios automatically computed by Medit had a median value of 77.16%, very close to the ideal value, and a relatively balanced patient division against this threshold (Figure 9b). The anterior Bolton ratios provided by dentOne had a median value of 78.15%, which was above the 77.20% threshold, with an intermediate dispersion, suggesting a relatively low excess of tooth substance in the mandibular arch compared to the jaw (Figure 9b).

The values of the total and anterior Bolton ratio are shown in Table 2.

The differences between the values of the manually measured ratios and those provided by the dentOne application were statistically analyzed using the Mann–Whitney test. The distributions of values were similar for both groups, when evaluated visually, and the result was not statistically significant, U = 51, z = −0.076, *p* = 0.940.

The differences between the values of manually measured anterior Bolton ratios and those provided by software applications were statistically analyzed using the Kruskal–Wallis test. The Bolton value distributions were similar for all three groups, and were evaluated by visual inspection using a boxplot graph. The median values of the Bolton ratios were not statistically significantly different between the three sets, χ^2^(3) = 0.188, *p* = 0.910 (Table 2).

**Figure 10:**
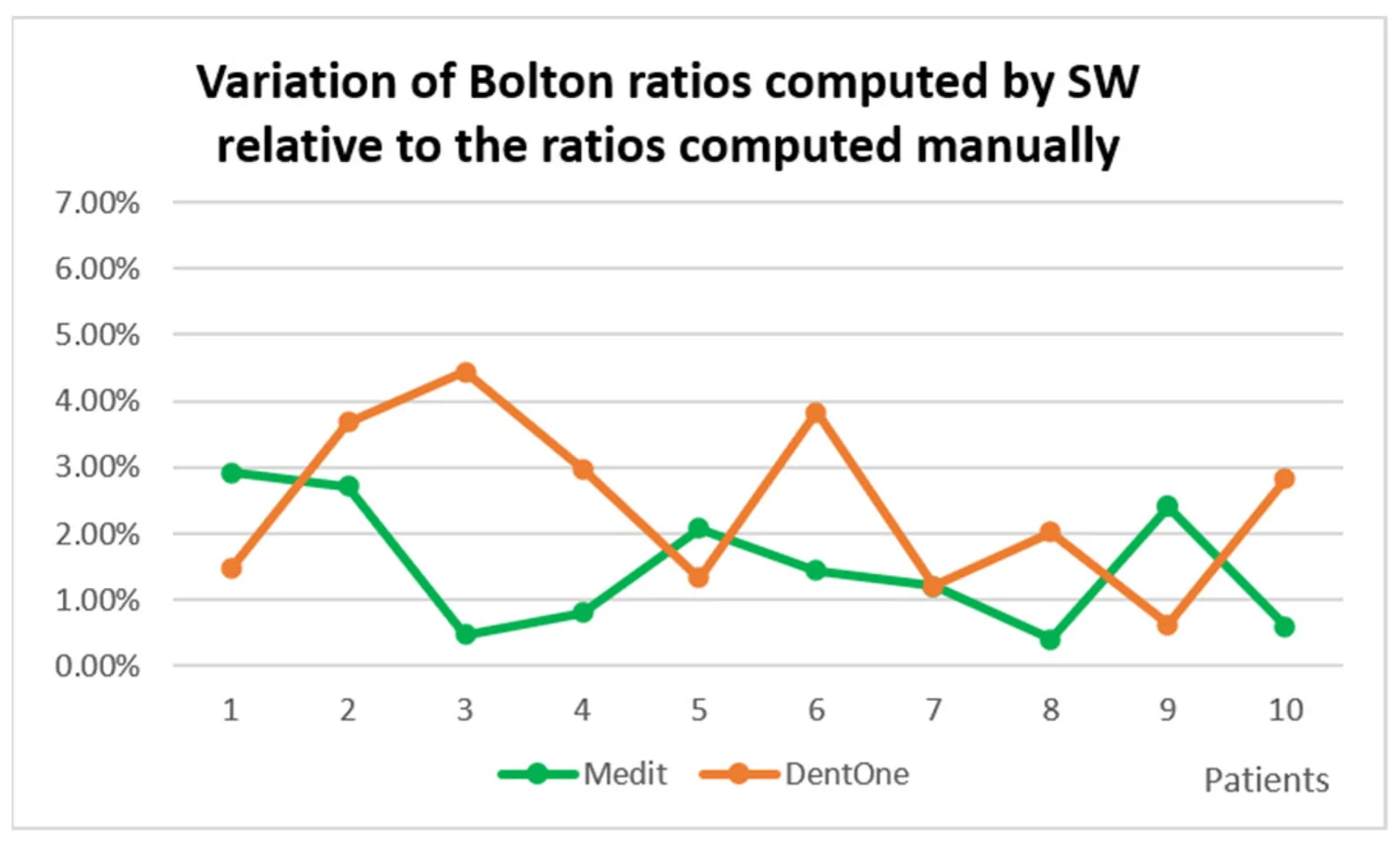
Variation in Bolton values computed by Medit Ortho Simulation and dentOne, relative to the ratio manually computed.

The absolute variation in the automated anterior Bolton values (Medit Ortho Simulation and dentOne) relative to manual measurements is shown in Figure 10, which highlights the differences in consistency between the two digital systems analyzed. The Medit Ortho Simulation evolution shows values relatively close to manual measurements, with a variation that is basically kept in the range of 1–3%, practically with the tendency to preserve the real dental proportions. The Bolton values provided by dentOne indicated a slightly higher global fluctuation, ranging between 0.63% and 4.44%. The variation in the two applications relative to manual measurements was statistically analyzed using the Wilcoxon signed-rank test, but no significant differences were determined between Medit Ortho Simulation (median change 1.76%) and dentOne (median change 2.42%), z = 0.652, *p* = 0.515.

### 3.2. Analysis of Individual Tooth Movements—Medit Ortho Simulation vs. dentOne

For the individual movements of the patients’ teeth, numerical and imaging comparisons were made between the results of the orthodontic treatments proposed by Medit Ortho Simulation (reserving an implant space), dentOne—with space closure, and dentOne—with fixed adjacent teeth and implicitly reserved implant space.

#### 3.2.1. Translational Motion Analysis

**Figure 11:**
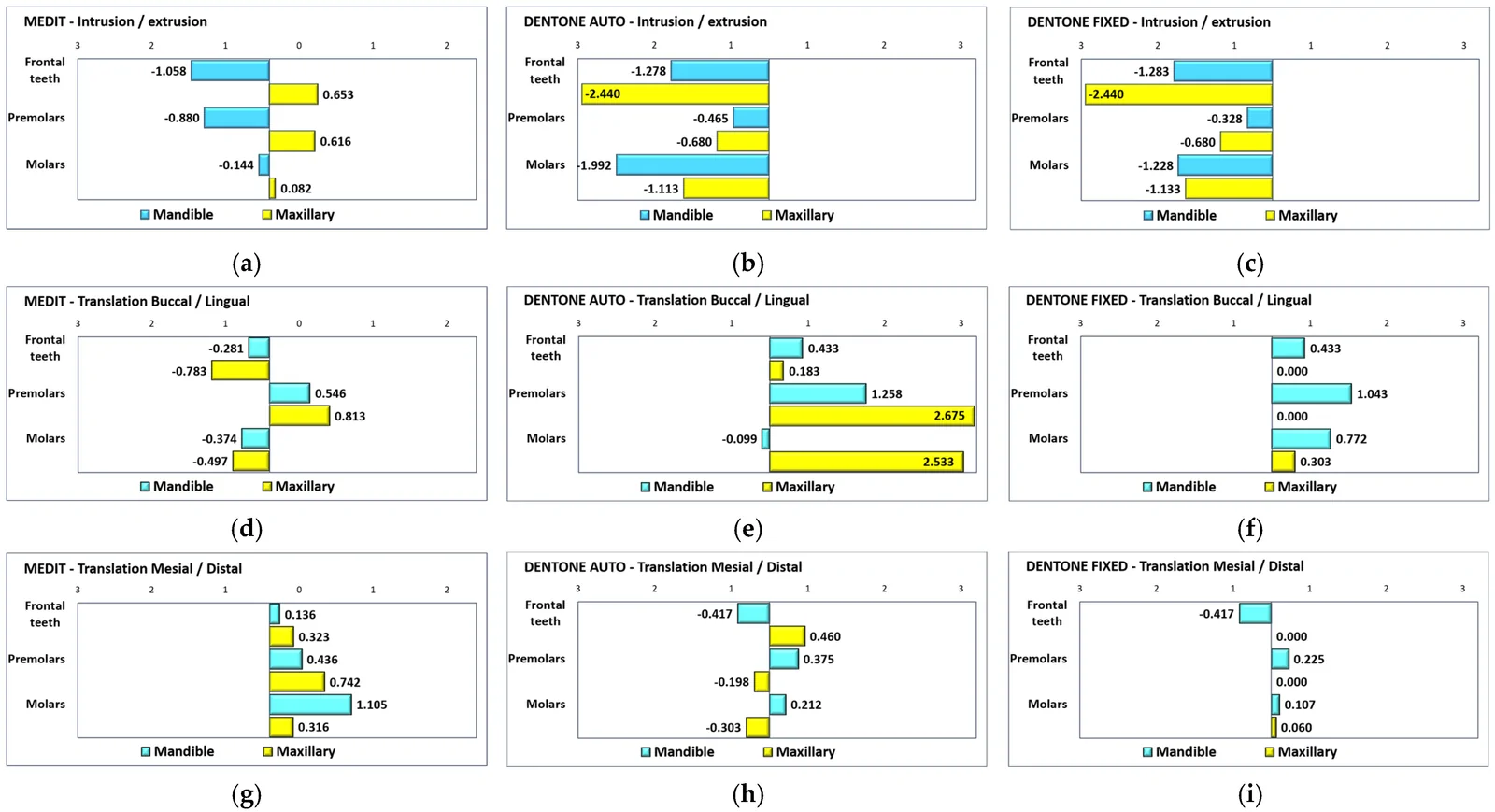
Graphical representation of the average linear movements of the teeth groups: (**a**–**c**) intrusion/extrusion; (**d**–**f**) buccal/lingual; (**g**–**i**) mesial/distal.

The comparative analysis of the intrusion/extrusion movements identified by the three software applications revealed variations between the values provided, both regarding the direction of movement of the teeth and the amplitude of their movements (Figure 11a–c). The Medit Ortho Simulation application suggested a mandibular intrusion movement for all three dental groups but also a maxillary extrusion with moderate values. Basically, according to Medit Ortho Simulation, the two arches must move in opposite directions but with decreasing intensity. The dentOne software application, for both dentOne Auto and Fixed variants, suggested a generalized intrusion movement, applicable both to the maxilla and mandible, with higher values planned for the front tooth group, moderate amplitudes for the premolar group, and intrusion movements for the molar group. These values indicated that the differences between the two versions of the dentOne application were minimal, especially when observing teeth near the implant area. Overall, Medit Ortho Simulation indicated a vertical divergence between the two arches, and dentOne described a synchronous, predominantly intrusive vertical displacement.

Comparative analysis of buccal/lingual displacements indicated by SW applications highlighted differences in the direction and amplitude of planned movements (Figure 11d–f). Medit Ortho Simulation suggested a compound movement, with alternating movements at the buccal and lingual levels. The groups of front teeth on both arches showed lingual displacement, the premolars showed a buccal displacement, and the molars showed a lingual movement with moderate amplitudes. The most pronounced evolution suggested was identified for dentOne Auto, which indicated displacements of 2.675 mm and 2.533 mm for the premolars and molars in the maxilla, in order to completely close the edentulous space, but also displacements with lower amplitudes at the level of the premolar and mandibular molar groups. The groups of front teeth were kept relatively in their initial position, with buccal translation movements with reduced amplitudes. The plan suggested by dentOne Fixed presented low and moderate movement variations by maintaining the edentulous space and the fixed position of the teeth adjacent to the space. Thus, buccal translations were moderate for all dental groups, and maxilla movements were reduced in amplitude or even non-existent, reducing inter-arch differences.

The comparison of the mesial–distal translational movements identified by the three applications reflected differences between the suggested treatment plans (Figure 11g–i). Medit Ortho Simulation described a predominantly mesial movement for all groups of teeth located on both arches, with amplitudes characterized by posterior progressive growth. The dentOne Auto had the greatest variability in terms of direction and amplitude of movement, alternating mesial and distal displacements. At the mandible level, the groups of front teeth and molars showed a distal movement, unlike the premolars, which had a movement with a similar amplitude but with a mesial direction. At the level of the maxilla, the movements were reversed, being mesial for the front teeth and molars and distal for the premolars, with similar amplitudes for the molars and premolars but higher for the front teeth. Conversely, dentOne Fixed, while preserving the edentulous space, showed small variations close to the initial state; the movement was minimal at the maxillary level, with a very low variation in amplitude only for the molar group. At the mandibular level, translational movements had moderate amplitudes, distal to the frontal teeth group and mesial to the premolar and molar groups. Overall, Medit Ortho Simulation suggested a progressive mesial displacement, and dentOne suggested alternating mesial/distal movements for both versions of algorithms, with higher amplitudes in the case of the Auto version.

#### 3.2.2. Analysis of Rotational Motion

**Figure 12:**
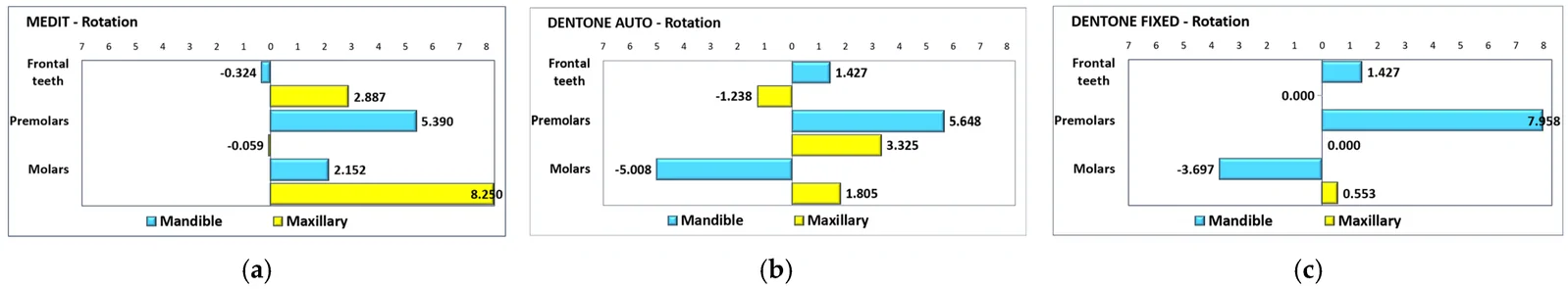

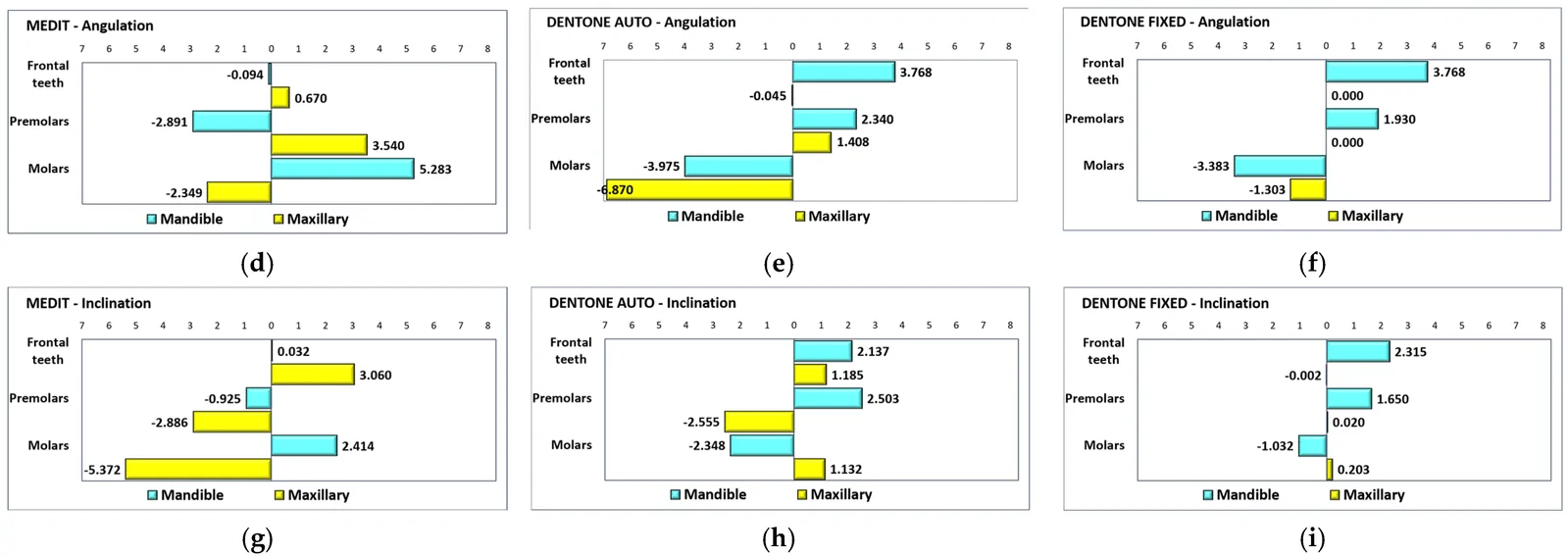
Graphical representation of the average rotational movements of the groups of teeth: (**a**–**c**) rotation; (**d**–**f**) angulation; (**g**–**i**) inclination.

The comparative analysis of the dental rotation movements revealed marked differences between the three planning algorithms, both in terms of the direction of rotation and its amplitude (Figure 12a–c). Medit Ortho Simulation planned a progressive increase in rotations in the maxilla, with lower values for the group of front teeth and higher values for the premolars and molars, while at the mandible level, the rotations were reduced or moderate in amplitude. The dentOne Auto exhibited opposite rotational movements between the arches for the front teeth group, with relatively similar amplitudes. The group of premolars was the only one that showed rotations in the same direction for both arches. The movement of the molar groups presented negative mandibular rotation, with a pronounced amplitude in association with a positive rotation at the level of the maxilla, and a reduced amplitude with respect to the mandible. The plan suggested by dentOne Fixed almost completely reduced the rotational movements in the maxilla, keeping only a relatively low value for the rotation of the molars. At the mandible level, pronounced values were maintained for the positive rotation of the premolars and negative rotation of the molars.

The comparison of the dental movements planned by each algorithm revealed clear differences between the three software applications used (Figure 12d–f). Medit Ortho Simulation maintained the increasing pattern of angulations at the level of the maxilla, with reduced amplitudes for the group of frontal teeth and more pronounced for the group of molars. At the mandible level, Medit Ortho Simulation suggested negative angulations for all groups of teeth, with low or moderate values. The dentOne Auto exhibited the greatest variations in motion, with pronounced negative angulations at the molars, defined for both arches, and moderate positive angulations at the premolar groups on both arches. The groups of frontal teeth had a different angulation between the arches, with a higher positive value for the maxilla but a very small negative value for the mandible. Considering the fact that dentOne Fixed oriented the entire treatment plan to maintain the edentulous space and fixed positions of the adjacent teeth, the algorithm kept the maxilla state as close as possible to the initial one by defining a low angulation value only for the molar group; instead, it reconfigured the mandibular context by suggesting moderate positive angulations for the front tooth and molar groups, as well as moderate negative angulations for the group of molars.

The different evolution of the three applications was also confirmed by the comparative analysis of dental inclinations in terms of direction and amplitude (Figure 12g–i). Medit Ortho Simulation suggested a completely different set of movements between the two dental arches; at the level of the mandible, minimum inclinations were provided for the groups of front teeth and premolars (with a positive and negative average, respectively), but at the level of the maxilla, the movements were much more extensive, especially for the group of molars, presenting the maximum difference between the arches. The dentOne Auto planned positive inclinations for the front teeth, with relatively similar values. The groups of premolars and molars had opposite inclinations between the two arches, with positive values for the premolars in the maxilla and negative values for the premolars in the mandible, as well as inverted values for the molars, which had negative inclinations in the maxilla and positive ones in the mandible. The dentOne Fixed algorithm showed the lowest tilt movement overall, with the maxillary context being kept almost identical to the original one, and with low positive inclinations for the front and premolar tooth groups and negative for the molar group.

The orthodontist’s analysis is presented comparatively for the three setups in the table below (Table 3). For the evaluation of the three simulations, the orthodontist used a scale from zero to 10. The zero scale is the simulation that was farthest from the orthodontist treatment plan, and the 10 scale is the simulation closest to the orthodontist treatment plan.

**Figure 13:**
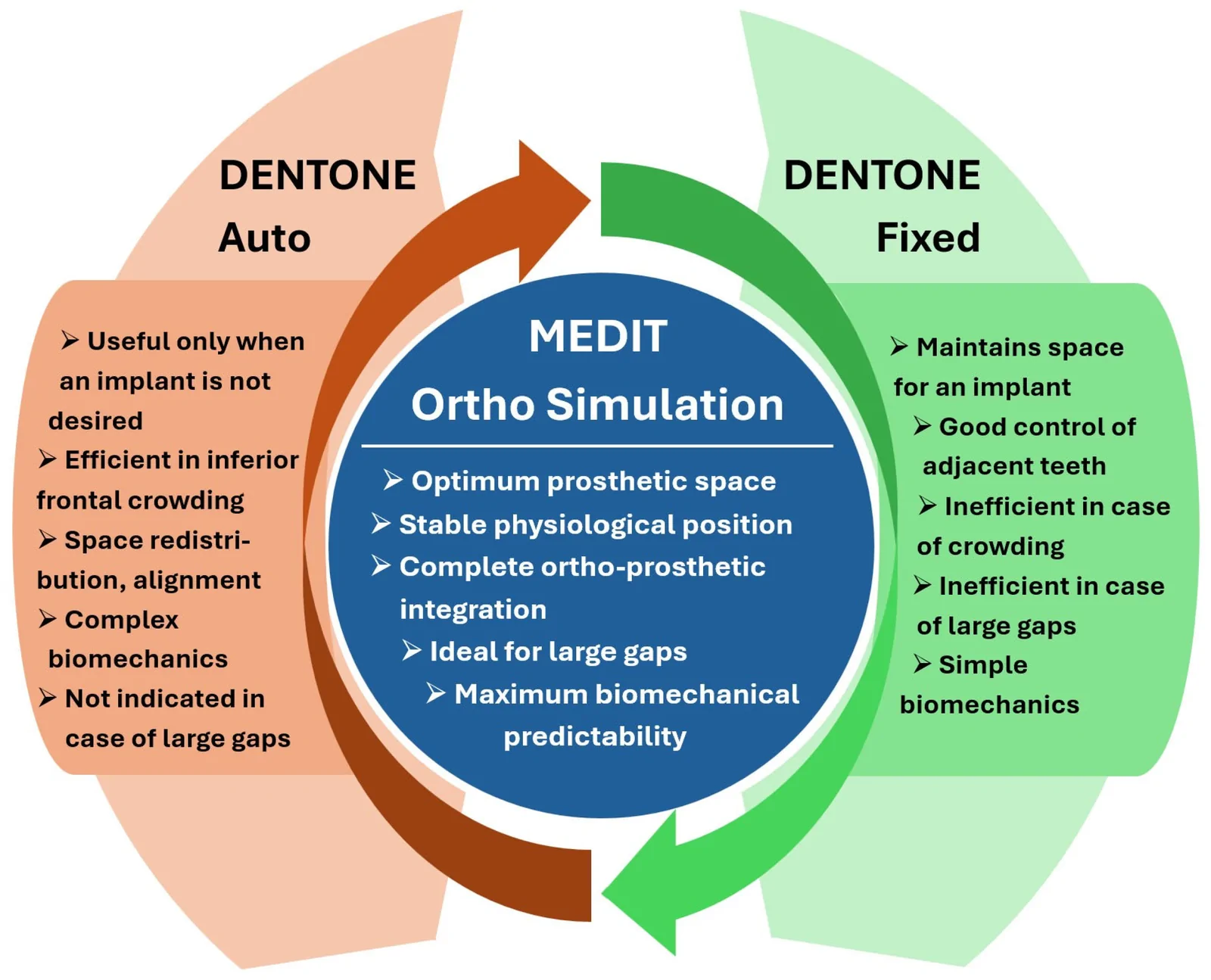
Comparative synthesis of the three simulation tools.

Based on the ten clinical cases, Figure 13 emphasizes a comparative synthesis of the three studied digital orthodontic tools—Medit Ortho Simulation, dentOne Auto, and dentOne Fixed—highlighting their clinical applicability, predictability, and biomechanics, as well as strengths and limitations for each software simulator.

## 4. Discussion

The continuous development of digital technologies and the integration of artificial intelligence into orthodontic practice have significantly altered the way diagnoses and treatment planning are carried out [2]. The present study comparatively analyzed the orthodontic treatment plans proposed by the Medit Ortho Simulation and Diorco dentOne software in the situation of reduced lateral edentulousness. As far as we know, it is the first study in this category. The Ortho Simulation software included in its planning the presence of a simulated implant through a cylinder with standard dimensions for an implant, which requires the preservation of a certain space in relation to the neighboring teeth, thus directing the movements of the other teeth in the three planes. The dentOne software proposed closing the space through orthodontic treatment, which led to extensive changes in the positions of teeth, while in the Autoalign variant, it did not allow the configuration of the space for an implant. In order to preserve the implant-prosthetic space, the teeth neighboring the gap were fixed; however, also in this case, the amplitude of the dental displacements was higher than in the case of Ortho Simulation’s treatment plans. The sharp dispersion of the variance of the Bolton ratios compared to manual measurements indicated a greater sensitivity of the dentOne system to individual morphological peculiarities and to the quality of the scan or the way of filling in the missing teeth, generating a variability that can influence the accuracy of the orthodontic diagnosis. Comparatively, Ortho Simulation positions itself as a tool with superior reproducibility. The evaluation of the three orthodontic therapeutic plans by an orthodontist showed that the Ortho Simulation software produced results closer to the planning of the orthodontist’s preimplantation orthodontic therapy compared to dentOne.

Using automatic digital configurations contributes to the efficiency of the clinical workflow, offering the possibility of rapid simulation of the therapeutic outcome and facilitating the clinician’s decision-making process [2]. In addition, these tools allow for clearer communication with the patient by visually presenting the anticipated changes during treatment [23].

However, although automated systems have demonstrated promising results, their performance is still limited compared to the planning carried out by an experienced orthodontist. The most obvious differences occur in complex clinical cases, especially when the treatment involves tooth extractions, rigorous anchorage control, or high-amplitude tooth movements [24]. In these situations, the automatically generated configurations fail to accurately reproduce the biomechanical objectives pursued by the clinician, which can lead to discrepancies between the virtual simulation and the desired clinical outcome [2,24].

An important factor influencing the accuracy of digital configurations is the process of tooth segmentation. Numerous studies have highlighted the difficulties encountered by current algorithms in accurately identifying dental boundaries and longitudinal axes, especially in the presence of severe crowding, dental restorations, or individual morphological changes [25]. These limitations can cause errors in positioning, rotation, and tooth inclination, thus affecting the realism and predictability of orthodontic simulations [26]. As a result, automated segmentation continues to be one of the main challenges in the development of fully automated digital systems [5].

The analyzed results also demonstrated the existence of significant differences between the software platforms used for the virtual configuration of preimplant orthodontic treatment. Even in situations where the same values of dental movements are introduced, the end positions generated by different programs can vary considerably [27]. These differences are influenced by the specific algorithms of each software, the segmentation methods used, and the way in which the centers of rotation of the teeth are calculated. Therefore, the choice of digital platform can have a direct impact on the outcome of the simulation and on the predictability of treatment [5,27]. The differences in movement between the Ortho Simulation and both variants of dentOne have direct implications for the clinical interpretation of treatment plans, as they can influence assessments of occlusion, tooth stability, and overall treatment planning that depend on the accuracy of vertical measurements.

In addition to its use in the simulation of dental movements, artificial intelligence has expanded its applicability to multiple stages of orthodontic diagnosis [8]. Modern algorithms are used for the automatic identification of cephalometric landmarks, assessment of skeletal maturation, classification of malocclusions, analysis of radiological images, and support in the therapeutic planning process. Recent studies have indicated that these systems can achieve high levels of accuracy and reproducibility, significantly reducing the time required for conventionally performed analyses [28]. However, the accuracy of the results depends directly on the quality of the data used to train the algorithms and on their permanent validation in relation to the evaluation carried out by specialists [29].

An important limitation of most systems currently available is the lack of information regarding root anatomy. And in the case of the present study, no radiological data on the positions of the teeth roots were used. The simulations were mainly carried out on the basis of digital models of dental crowns, without integrating the position and orientation of the roots. This approach can reduce the accuracy of estimating teeth movements and influence biomechanical treatment planning [29].

For this reason, the integration of images obtained by cone beam computed tomography (CBCT) is one of the most important directions for the development of digital orthodontics, offering the possibility of building virtual models much closer to clinical reality [30]. However, this overlap of digital information is a challenge for manufacturers of orthodontic treatment simulation platforms, requiring efforts to make software compatible. It is necessary to mention that, currently, the Medit Link platform in which the Medit applications are found has completely given up the Ortho Simulation application, replacing it with the Medit Orthodontic Suite starting from June 2026. This application allows the overlapping of the digital model obtained by intraoral scanning with cephalometry, which offers the advantage of orthodontic treatment planning by taking into account the positions of dental roots.

The literature also highlights the benefits of using digital technologies in interdisciplinary treatments [31]. The association of data from intraoral scans, CBCT images, and three-dimensional planning software facilitates collaboration between orthodontics, implantology, and dental prosthodontics [32]. This approach allows for a more complete evaluation of clinical cases and contributes to increasing the predictability of therapeutic outcomes by simultaneously integrating functional, aesthetic, and biological considerations [32].

Overall, researchers have supported the idea that artificial intelligence and digital technologies have since become indispensable tools in modern orthodontics [33]. They offer important advantages in terms of efficiency, standardization, and predictability of treatment. However, the current level of technological development does not allow for the complete replacement of clinicians’ expertise [8]. Artificial intelligence simulation permit the visualization of results of orthodontic treatments before treatment decisions, which is useful for both the orthodontist and the general dentist in the oral implant-prosthetic rehabilitation approach. Interpreting the results, adapting the treatment plan to the particularities of each patient, and validating therapeutic decisions remain fundamental responsibilities of the orthodontist. Consequently, artificial intelligence must be seen as a complementary tool that supports the clinical process and not as a substitute for professional judgment [34]. Due to the differences between the simulations produced by the software, the doctor must be well-acquainted with their characteristics to use them successfully.

The limitations of the present study are represented by the small number of cases and the absence of overlapping radiological images of the roots. For the future, it is desirable to analyze digital simulation platforms for preimplant orthodontic treatment planning, in which they are used in addition to digital models and radiological images of dental roots.

## 5. Conclusions

Software applications for simulating orthodontic treatment in cases of implant-prosthetic rehabilitation are necessary tools for the orthodontist, general dentist, implantologist, and prosthodontist involved in a patient’s oral rehabilitation process. The results of the study showed that the simulations of preimplantation orthodontic treatments were significantly different between the two software and the three corresponding orthodontic setups. Thus, the Medit Ortho Simulation application was considered by the orthodontist as the one that provided the most appropriate simulations of an orthodontist’s thinking. The dentOne Autoalign application simulated the closure of potential prosthetic space, which did not allow for the positioning of an implant in the edentulous space. This could only be achieved in this application by fixing the teeth adjacent to the edentulous gap. Both simulations of preimplant orthodontic treatment with dentOne used much larger teeth movements compared to Ortho Simulation. It is important to emphasize that doctors who want to use such tools must be aware that, since simulations differ from one platform to the next, they should choose the tool closest to their treatment philosophy so that the therapeutic proposals maximize efficiency with minimal adverse effects.

## Figures and Tables

**Table 1:** Cases included in the analysis, including details of edentulousness.

Case	Maxillary Tooth Loss	Mandibular Tooth Loss
1	17	36
2	-	36
3	-	36
4	-	36
5	-	36
6	-	36
7	-	36
8	15, 16	35, 36
9	-	36
10	-	36

**Table 2:** Bolton ratio metrics for each app used.

Case	Total Bolton Ratio			Anterior Bolton Ratio			Anterior—Variation	
	Initial *	Medit **	dentOne **	Initial *	Medit **	dentOne **	Medit **	dentOne **
1	102.35%	-	97.83%	80.76%	83.68%	82.23%	2.92%	1.47%
2	91.17%	-	90.44%	77.98%	80.69%	81.66%	2.71%	3.68%
3	88.23%	-	89.81%	71.31%	71.78%	75.75%	0.47%	4.44%
4	90.19%	-	90.15%	76.16%	76.97%	79.13%	0.81%	2.97%
5	92.48%	-	92.09%	76.25%	74.18%	74.93%	2.07%	1.32%
6	83.20%	-	89.06%	71.59%	70.15%	75.42%	1.44%	3.83%
7	93.60%	-	93.28%	77.92%	76.71%	79.13%	1.21%	1.21%
8	87.93%	-	85.20%	78.35%	94.05%	80.37%	15.70%	2.02%
9	90.25%	-	93.51%	76.54%	78.94%	77.17%	2.40%	0.63%
10	92.69%	-	91.06%	77.95%	77.35%	75.13%	0.60%	2.82%
Mean ± SD	91.21 ± 4.94	-	91.24 ± 3.31	76.48 ± 2.97	78.45 ± 6.78	78.09 ± 2.78	2.11 ± 1.96	2.44 ± 1.29
Median	90.71	-	90.75	77.23	77.16	78.15	1.60	2.42
*p*		0.940 ^#^			0.910 ^##^		0.086 ^###^	

* Manually measured values. ** Values computed using SW applications. ^#^ Mann–Whitney. ^##^ Kruskal–Wallis. ^###^ Wilcoxon signed-rank.

**Table 3:** The evaluation made by the orthodontist on the three simulations (grade intervals 1–10).

Case	Grade and Arguments for Medit Ortho Simulation	Grade and Arguments for dentOne Auto Simulation	Grade and Arguments for dentOne Fixed Simulation
1	Grade 8 Anatomical restoration of the edentulous space, and of the dental anatomy of teeth affected by severe wear. Integration of implant treatment with prosthetic rehabilitation. Facilitation of the reconstruction of the vertical size of occlusion. Preservation of the physiological position of adjacent teeth and occlusal relationships.	Grade 1 Not suitable in this case since the edentulous gap corresponding to the consecutive absence of teeth 35 and 36 had a large mesial–distal diameter. Orthodontic closure of such a gap involves extensive dental displacement, difficult biomechanics, and an increased risk of alteration of the physiological positions of posterior teeth. These difficulties were accentuated by excessive dental wear and reduced coronary volume, which limits orthodontic control and treatment predictability. This variant was inferior to digitally planned implant-prosthetic treatment.	Grade 3 Limited therapeutic alternative in this situation, as the edentulous gap was significantly altered compared to the physiological size. Excessive dental wear and the significant reduction in coronary volume made orthodontic treatment more difficult, limiting the retention of brackets/attachments and the biomechanical control of dental movements.
2	Grade 10 Optimal prosthetic space for implant restoration. Preservation of the physiological positions of teeth 35, 37, and 38. Facilitation of the insertion of the implant in an optimal position. Total Bolton ratio (90.44%), which was very close to the ideal value and supported a stable occlusion. Best long-term functional, biological, and aesthetic predictability.	Grade 3 Could only be chosen if the main objective was to avoid the implant. In this case, the closure of the gap involved the important mesial movement of posterior mandibular teeth, with the need for rigorous control of anchorage, torque, and root parallelism. This solution involved complex biomechanics and could have altered the physiological positions of posterior teeth and the stability of the long-term occlusion.	Grade 7 Good therapeutic alternative, as it preserved the space intended for the implant and allowed relatively simple orthodontic biomechanics, with good control for the positions of adjacent teeth. Planning did not benefit from the full digital integration of orthodontic and prosthetic parameters provided by Medit analysis; following the loss of 36, teeth 37 and 38 underwent a tipping towards the mesial, altering the space for a future crown at the level of the 36 implant. Root parallelism also suffered through the mesial tilting of 37 and 38.
3	Grade 10 Optimal prosthetic space for implant restoration. Preservation of the physiological positions of adjacent teeth.	Grade 6 Advantageous if the main objective was to avoid the implant, and was supported by the existence of crowding in the lower frontal region, given that the space resulting from closing the gap could be used to align the arch. Orthodontic biomechanics remained complex and required a rigorous balance between the mesialization of teeth located distal to the breach and the distalization of teeth located mesial to the gap in order to maintain occlusal relationships and the correspondence of interincisive lines.	Grade 5 Not a very effective alternative in this situation, since maintaining the edentulous space did not contribute to solving the crowding in the lower frontal region. Complete alignment of the lower incisors would have required a marked proclination of the lower incisors or a limited interproximal reduction, depending on Bolton analysis.
4	Grade 10 Ideal prosthetic space for implant restoration.	Grade 4 Chosen if the main objective was to avoid the implant. In this case, the closure of the gap involved the important mesialization of posterior mandibular teeth, with the need for rigorous control of anchorage, torque, and root parallelism. Much more complex biomechanics that could alter the physiological position of the posterior teeth and the stability of the occlusion in the long term.	Grade 7 Effective therapeutic alternative, as it preserved the space intended for the implant and allowed relatively simple orthodontic biomechanics, with good control for the positions of adjacent teeth. Planning did not benefit from the full digital integration of orthodontic and prosthetic parameters provided by the Medit analysis; following the loss of 36, 37 underwent a tipping towards the mesial, altering the space for a future crown at the level of the 36 implant. Root parallelism also suffered through the mesial tipping of 37.
5	Grade 10 Optimal prosthetic space for implant restoration. Preservation of the physiological positions of adjacent teeth. Facilitation of the insertion of the implant in an ideal position. Values of the Bolton ratio were close to normal, which supported achieving a stable occlusion without major compromises. Best long-term functional and biological predictability.	Grade 3 Advantageous if the main objective was to avoid the implant. Orthodontic biomechanics was complex, and a perfect balance was required between the mesial displacement of teeth located distal to the gap and the distal displacement of teeth located mesial to the gap, with the latter being a particularly complex displacement, making it especially important to ensure the correspondence of interincisive lines.	Grade 7 Not a very effective alternative in this situation, as it planned a marked retrusion of the lower incisors to close the spaces resulting from the migration of teeth following the edentulousness of 36 for alignment of the lower frontal group, or the performance of additive techniques at the level of lower teeth within the limit of the Bolton ratio.
6	Grade 10 Optimal prosthetic space for implant restoration. Preservation of the physiological positions of adjacent teeth. Facilitation of the insertion of the implant in an ideal position. Values of the Bolton ratio were close to normal, which supported achieving a stable occlusion without major compromises.	Grade 6 Advantageous if the main objective was to avoid the implant; at the same time, it was supported by the existence of a dento-alveolar disharmony in the inferior anterior region. Orthodontic biomechanics was quite complex, and it required a perfect balance between the mesial displacement of teeth located distal to the gap and the distal displacement of teeth located mesial to the gap, making it especially important to ensure the correspondence of interincisive lines.	Grade 5 Not a very effective alternative in this situation, as it planned a sharp proclination of the lower incisors to create space for alignment of the inferior frontal group or limited interproximal reduction according to the Bolton analysis.
7	Grade 10 Optimal prosthetic space for implant restoration. Preservation of the physiological positions of adjacent teeth. Facilitation of the insertion of the implant in an ideal position. Values of the Bolton ratio were close to normal, which supported achieving a stable occlusion without major compromises. Best long-term functional and biological predictability.	Grade 6 Advantageous if the main objective was to avoid the implant; at the same time, it was supported by the existence of a dento-alveolar disharmony in the inferior anterior region. Orthodontic biomechanics were quite complex and required a perfect balance between the mesial displacement of teeth located distal to the gap and the distal displacement of teeth located mesial to the gap, making it especially important to ensure the correspondence of interincisive lines.	Grade 5 Not a very effective alternative in this situation, as it required a sharp proclination of the lower incisors to create space for alignment of the inferior frontal group or limited interproximal reduction according to the Bolton analysis.
8	Grade 10 Anatomical restoration of the four missing teeth. Preservation of the physiological positions of adjacent teeth and occlusal relationships. Facilitation of the insertion of implants in ideal positions and the realization of predictable prosthetic restorations. Bolton analysis allowed the identification and integration of any dento-dental discrepancies in the treatment plan. Best distribution of occlusal forces. Best long-term functional prognosis.	Grade 1 Not suitable in this case, as the edentulous gaps had considerable mid-distal diameters. Orthodontic closure of two spaces corresponding to the absences of the second premolar and six-year-old molar involved extensive dental displacements, difficult biomechanics, and an increased risk of alteration of the physiological positions of posterior teeth. Less predictable from a functional and biological point of view compared to implant-prosthetic treatment.	Grade 5 Ineffective therapeutic alternative in this situation, since the edentulous spaces were significantly altered by volume.
9	Grade 10 Adequate prosthetic space. Maintaining the physiological positions of adjacent teeth. Creating the ideal conditions for implant insertion. Bolton analysis values, close to normal, supported the achievement of a functional and stable restoration, without the need for significant compromises on occlusion.	Grade 3 A viable solution only when the main objective was to avoid implantation. It reduced the number of therapeutic steps, which involved extensive dental movements, more difficult biomechanical control, and lower functional predictability compared to the implant restoration option. Mesio-distal size of the edentulous gap was larger while, at the same time, 38 was present, making the orthodontic biomechanics necessary for this situation increasingly complex.	Grade 9 Preserves the space necessary for restoration and simplifies orthodontic biomechanics, but requires subsequent implant treatment.
10	Grade 10 Restored the physiological positions of adjacent teeth, creating an optimal prosthetic space for restoration. Allowed the implant to be inserted into an ideal position. Provided the best distribution of occlusal forces and the best long-term predictability. Values of the Bolton analysis were close to normal, which supported achieving a stable occlusion without major compromises.	Grade 3 Advantageous if the main objective was to avoid the implant. It involved the widest dental displacements and the most complex biomechanics, which made it less favorable when there was the possibility of a correct implant restoration.	Grade 9 Very good alternative when the purpose was just to maintain the space until the implant was inserted, serving as a simple and predictable approach.

## Data Availability

The data presented in this study are available on request from the corresponding authors due to privacy, legal, and ethical restrictions.
